# Epistemological beliefs and therapeutic health concepts of physiotherapy students and professionals

**DOI:** 10.1186/1472-6920-14-208

**Published:** 2014-10-01

**Authors:** Martina Bientzle, Ulrike Cress, Joachim Kimmerle

**Affiliations:** Knowledge Construction Lab, Knowledge Media Research Center, Schleichstr. 6, 72076 Tuebingen, Germany; Department of Psychology, University of Tuebingen, Schleichstr. 4, 72076 Tuebingen, Germany

**Keywords:** Epistemological beliefs, Therapeutic health concepts, Health knowledge, Training status

## Abstract

**Background:**

Health knowledge develops fast and includes a lot of ambiguous or tentative information. In their daily routine, both health care students and professionals continuously have to make judgments about the viability of health knowledge. People’s epistemological beliefs (EBs) and their therapeutic health concepts are factors that influence how they deal with health knowledge. However, very little is known about the occurrence of these factors at different stages of people’s career. The present study examines the EBs and therapeutic health concepts of physiotherapy students in their vocational training and the EBs and therapeutic health concepts of professionals.

**Methods:**

In a cross-sectional study physiotherapy students and professional physiotherapists filled in a questionnaire that measured their personal EBs about physiotherapy and medicine, as well as their biomedical and biopsychosocial therapeutic health concepts. We compared the participants’ EBs regarding both knowledge domains, and their therapeutic health concepts using paired samples t-tests. We also examined the differences between first-year students, advanced students, and professionals regarding their EBs and their therapeutic health concepts using ANOVAs.

**Results:**

Eighty-three students and 84 professionals participated in this study, 114/167 (68%) participants were female. EBs as well as therapeutic health concepts differed depending upon the participants’ training status. Professionals had more sophisticated EBs than students regarding both knowledge in physiotherapy (F(2, 164) = 6.74, P = 0.002, η^2^_p_ = 0.08) and knowledge in medicine (F(2, 164) = 5.93, P = 0.003, η^2^_p_ = 0.07). In addition, high values in a biopsychosocial therapeutic health concept already occurred in an early phase of training (F(2, 164) = 5.39, P = 0.005, η^2^_p_ = 0.06), whereas increased values in a biomedical concept did not occur until people’s professional life (F(2, 164) = 10.99, P < 0.001, η^2^_p_ = 0.12).

**Conclusions:**

The specificities of personal EBs and therapeutic health concepts in different stages of health care training have so far been insufficiently considered in medical education research. The current study has aimed to shed light on the occurrence of these concepts in students as compared to professionals. We point out implications of our findings for educational practice and make suggestions for future research.

## Background

In order to master their respective tasks, both health care students and professionals consistently have to make judgments about the viability of health knowledge. This holds true all the more as health knowledge is widespread and very comprehensive, develops fast, and includes a lot of ambiguous or tentative information [1, 2]. One factor that influences how people deal with health knowledge is their body of personal *epistemological beliefs* (EBs) [3, 4]. “Epistemological beliefs are the cognitions (i.e., understandings) individuals have on knowledge and knowing and determine how (new) knowledge is perceived and processed.” (p. 616) [5]. EBs are linked to core domains of education such as learning, motivation, reasoning, and academic performance [6–10]. It has been criticized, however, that to date they have been insufficiently investigated in educational [11] and especially medical education research [2, 5]. This is a shortcoming, because health care professionals are explicitly called upon for critical appraisal of health knowledge and medical literature—particularly since evidence-based medicine becomes increasingly significant in the course of their practice [12–15]. This applies both to the phase of their medical training and to their entire professional life.

Another factor that influences how people deal with health knowledge is their personal *therapeutic health concept* [16, 17] *.* This also plays an important role in the professional life of health care providers. A therapeutic health concept is a specific point of view about health and therapy. In the health care sector a *biomedical* therapeutic health concept can be differentiated from a *biopsychosocial* concept [18–20]. It was found that the therapeutic health concept in which health information is embedded influences how students deal with this information [16]. Additionally, the particular therapeutic health concept has an impact on attitude and therapeutic behavior of health care professionals [17]. As a whole, a therapeutic health concept can be considered as a meta-concept, which shapes the nature of health care professionals’ work. Thus, the development of a therapeutic health concept can be regarded as one part of the professionalization process [21, 22] that is supposed to begin during people’s training and continues throughout their professional life. However, very little is known about the therapeutic health concepts of people in training compared to that of professionals. The present analysis aims to contribute to a better understanding of EBs and therapeutic health concepts of physiotherapy students in comparison to professional physiotherapists.

### Health-related EBs

EBs describe people’s subjective understanding about the nature of knowledge and the process of knowing [4, 5, 10]. A continuum is assumed from simple EBs (considering knowledge as certain and absolute) to sophisticated EBs (considering knowledge as variable, constructed, and tentative) [23]. A fundamental and topical question in EB research is whether EBs are domain general or domain specific [6, 23–25]. More evidence is needed about whether people’s particular beliefs about knowledge apply in the same way to diverse types of disciplines (domain-general EBs) or differ among various disciplines, with more sophisticated EBs regarding one domain compared to another (domain-specific EBs). In the medical realm, people’s EBs also have to be considered in the context of particular domains: Health care professionals frequently work in interdisciplinary teams, and the question arises how they evaluate health knowledge in their own discipline (e.g., physiotherapy) in comparison with adjacent disciplines. Since people have a more elaborate overview of their own discipline, we assume that beliefs about knowledge of one’s own discipline (here: physiotherapy) are more sophisticated than beliefs about knowledge of an adjacent discipline (medicine). (Hypothesis 1: main effect of domain on health-related EBs).

Regarding the development of EBs toward a higher level of sophistication, it was found that critical appraisal of information and dealing with ill-structured problems [26] have a positive influence on the development of personal EBs [27]. In their daily routine, health care professionals are constantly confronted with a variety of challenges: The process of medical decision-making, for example, requires the ability to match diverse information from various sources and of varying quality. Health care professionals have to integrate diagnostic findings, the current state of scientific evidence, the social context, and patients’ individual characteristics to make an adequate treatment decision. In the progression of their training and their professional life this holds true to an increasing degree. Hence we assume that professionals have more sophisticated health-related EBs than students, and advanced students have more sophisticated health-related EBs than first-year students (Hypothesis 2: main effect of training status on health-related EBs).

### Therapeutic health concepts

The International Classification of Diseases (ICD) [28] and the International Classification of Functioning, Disability and Health (ICF) [29] are concurrent international references and can be jointly used [30] in the health care sector. In these classification systems two different therapeutic health concepts became manifest: The ICD is based on a biomedical (*bm*) understanding of diagnostic and therapeutic procedures, whereas the usage of the ICF is consistent with a biopsychosocial (*bps*) conception. While the ICD is prevalent in conventional medicine, the ICF is increasingly used in physiotherapy [31]. Accordingly, the *bps* therapeutic health concept is common and popular in physiotherapy [16, 32, 33]. Thus, we assume that physiotherapists place higher value in a *bps* than in a *bm* concept (Hypothesis 3).

The development of a therapeutic health concept can be regarded as one part of the professionalization process [21, 22], and it influences people’s individual view of their profession. Thus, the therapeutic health concept can be interpreted as one aspect of the occupational identity of health care professionals. This identity develops in part through interactions with colleagues [34]. Therefore, we assume that the therapeutic health concept differs according to the experience levels of professionals, advanced students and first-year students. That is, we suppose that the therapeutic health concept that is characteristic of a particular profession is more prominent for professionals than for students, as well as more prominent for advanced students than for first-year students. Accordingly, we hypothesize that professional physiotherapists have a more pronounced *bps* concept than physiotherapy students, and that advanced students have a more pronounced *bps* concept than first-year students (Hypothesis 4a: main effect of training status on the *bps* concept).

Analogously, people attribute less relevance to concepts that are not compatible with their own social identity [35]. This should be less distinctive for students who have not yet developed a strong professional identity. Consequently, we assume that professional physiotherapists have a less pronounced *bm* concept than physiotherapy students, and that advanced students have a less pronounced *bm* concept than first-year students (Hypothesis 4b: main effect of training status on the *bm* concept).

## Methods

### Participants

A total of 167 participants took part in the investigation and filled in a questionnaire that measured their personal EBs about physiotherapy and medicine, as well as their *bm* and *bps* therapeutic health concepts. One hundred and fourteen participants were women, 52 were men, and one did not indicate his/her gender. Eighty-four participants were students of a school of physiotherapy (PT Academy) in the first to third (senior) year of their vocational training, 83 participants were professional physiotherapists. Eighteen first-year students were women and 11 were men. Forty advanced students were women and 15 were men. Fifty-six professionals were women, 26 were men, and one did not indicate his/her gender. So the gender distribution was similar in all groups, with a ratio of females varying between 62% and 73%. But of course, students and professionals differed in age: 95% of the students were between 21 and 30 years old, only 5% were between 31 and 40. In the group of professionals, in contrast, only 29% were between 21 and 30 years old, 28% were between 31 and 40, 27% were between 41 and 50, and 15% were older than 51 years. While all students were recruited from the same school of physiotherapy, the professionals were trained at various schools and were also diverse with regard to their current working environment: 26 worked in a hospital (19 in an emergency hospital, seven in a rehabilitation center), 33 were employed in a physiotherapy practice, 25 were self-employed in a physiotherapy practice, and 18 worked as lecturers (note that multiple answers were possible here, as several participants worked part-time in various jobs).

### Measurements

In order to measure participants’ health-related EBs, we used the Connotative Aspects of Epistemological Beliefs (CAEB) scale [23]. This semantic differential can be easily adapted to different content areas and is sufficiently sensitive to distinguish among domains [23]. The participants were asked to judge the knowledge in the domains of medicine and physiotherapy on seven-point semantic differential scales. Each domain was described with the same 17 items (pairs of adjectives). The CAEB comprises two factors (sub-scales). One factor is called *texture* and refers to the judgment of the structure and accuracy of knowledge. It is measured by items that consist of adjective pairs like “sorted – unsorted” or “absolute – relative”, with “sorted” and “absolute” representing simple and “unsorted” and “relative” representing sophisticated EBs. The other factor is called *variability* and refers to the supposed stability and dynamics of knowledge. It is measured by items that consist of adjective pairs like “static – dynamic” or “stable – unstable”, with “static” and “stable” representing simple and “dynamic” and “unstable” representing sophisticated EBs (see Table 1).

**Table 1:** **Connotative Aspects of Epistemological Beliefs (CAEB) scale (17 items version)**

Knowledge in the domain of physiotherapy [or medicine respectively] is …			
1	stable	□□□□□□□	unstable
2*	objective	□□□□□□□	subjective
3*	confirmable	□□□□□□□	unconfirmable
4^r^	dynamic	□□□□□□□	static
5*	superficial	□□□□□□□	profound
6^r^	temporary	□□□□□□□	everlasting
7*	exact	□□□□□□□	vague
8*	absolute	□□□□□□□	relative
9*	sorted	□□□□□□□	unsorted
10*	precise	□□□□□□□	imprecise
11^r^	flexible	□□□□□□□	inflexible
12*	definite	□□□□□□□	ambiguous
13*^r^	negotiated	□□□□□□□	discovered
14*	structured	□□□□□□□	unstructured
15	completed	□□□□□□□	uncompleted
16^r^	refutable	□□□□□□□	irrefutable
17^r^	open	□□□□□□□	closed

Seven-point semantic differential scales for measuring EBs about physiotherapy and medicine; items of the texture subscale are marked with an asterisk (*); reversely coded items are marked with a superscript ‘r’.

We captured the *bm* and *bps* concepts following the procedure presented by [16] (see Table 2): Participants were asked to rate the importance of five typical *bm* terms (e.g., ‘diagnosis’) and five typical *bps* concepts (e.g., ‘functionality’) on a six-point Likert scale ranging from 1 (not important) to 6 (very important).

**Table 2:** **Therapeutic health concepts scale**

How important are the following subjects for your therapeutic thinking and acting?	
1	Functionality
2*	Diagnosis
3*	Science
4*	Evidence-based methods
5	Limited activity of a patient
6*	Standardized tests
7	Limited participation of a patient (in the social environment)
8*	Medical guidelines
9	Mental health of a patient
10	Requirements of the patient’s everyday life

Six-point Likert scales for measuring the *bm* and *bps* concepts; bm items are marked with an asterisk (*).

### Statistical analysis

We performed data analysis using IBM SPSS 20.0 for Windows [36]. Internal consistencies were determined for all scales. We calculated Pearson product–moment correlations to analyze the relationship of EBs and therapeutic health concepts as well as the relationship of EBs regarding medicine and physiotherapy. For testing the hypotheses the level of significance was set at P < 0.050. Paired samples t-tests were performed to determine differences between EBs regarding the different knowledge domains and to test differences between the *bm* and the *bps* concepts. Moreover, we calculated ANOVAs to examine the differences between students and professionals regarding their EBs and their therapeutic health concepts. To take into account that three groups (first-year students, advanced students, and professionals) were compared, we calculated contrast analyses. In addition, we calculated Levene’s tests to examine homogeneity of the variances of the groups. We report all data as means (M) ± standard deviations. Cohen’s d and η^2^p are reported as effect sizes of mean differences.

### Ethics statement

This research was performed in accordance with the Declaration of Helsinki. The PT Academy’s administration provided ethical approval regarding participation of its students (due to legal specifications, the school administration was responsible for checking and approving the participation of its students). Regarding the other participants this study had full approval by the ethics committee of the Knowledge Media Research Center (approval number: KMRC-LEK 2013/035). All students and professionals participated voluntarily and anonymously. They gave informed consent and were informed about privacy protection, their right to terminate participation at any time without disadvantages, and about the general purpose of the study.

## Results

Internal consistency was good for the CAEB factor *texture* in both domains (α_*phys*_ = 0.75; α_*med*_ = 0.75) but poor for the factor *variability* (α_*phys*_ = 0.57; α_*med*_ = 0.56). Due to this psychometric shortcoming, the variability sub-scale had to be excluded from further analyses. Both health concepts sub-scales showed acceptable internal consistencies (α_*bm*_ = 0.69; α_*bps*_ = 0.66) and were independent from each other (r = 0.07, P = 0.399).

We analyzed whether EBs and therapeutic health concepts were related to each other. Pearson product–moment correlations indicated that both types of therapeutic health concepts and EBs for both domains were completely independent from each other (r_*bm, EBmed*_ = -0.03, P = 0.701; r_*bm, EBphys*_ = 0.00, P = 0.962; r_*bps, EBmed*_ = 0.08, P = 0.288; r_*bps, EBphys*_ = 0.10, P = 0.193). Accordingly, we address the findings regarding EBs and therapeutic health concepts in two separate sections.

### Health-related EBs

Hypothesis 1 assumed that physiotherapists’ health-related EBs were more sophisticated regarding knowledge in their own discipline compared to knowledge in an adjacent discipline. This assumption was supported by the data. The participants had less sophisticated EBs regarding knowledge in medicine (M = 3.53 ± 0.78) than regarding knowledge in physiotherapy (M = 3.72 ± 0.89; t(165) = -3.03, P = 0.003, d_z_ = 0.24). At the same time significant correlations occurred between the evaluation of knowledge in medicine and physiotherapy (r = 0.53, P < 0.001). This indicates that the EBs regarding both domains have some features in common, despite their differences.

Moreover, hypothesis 2 stated a main effect of training status (first-year students vs. advanced students vs. professionals) on health-related EBs. This assumption was predominantly supported by the data (see Table 3). We found significant differences between the EBs regarding knowledge in physiotherapy (F(2, 164) = 6.74, P = 0.002, η^2^_p_ = 0.08) and knowledge in medicine (F(2, 164) = 5.93, P = 0.003, η^2^_p_ = 0.07) depending on training status. The contrast analysis revealed that the comparison of the first year students with professionals was significant for EBs regarding knowledge in physiotherapy (P = 0.001) and regarding knowledge in medicine (P = 0.001). The comparison of the first-year students with advanced students was also significant for EBs regarding knowledge in physiotherapy (P = 0.035) and for EBs regarding knowledge in medicine (P = 0.028). Finally, the comparison of the advanced students with professionals was significant for EBs regarding knowledge in physiotherapy (P = 0.025), but not for EBs regarding knowledge in medicine (P = 0.258).

**Table 3:** **Health-related EBs**

EBs	EBs regarding knowledge in physiotherapy	EBs regarding knowledge in medicine
Training status		
First-year students	M = 3.30 ± 0.63	M = 3.11 ± 0.66
Advanced students	M = 3.61 ± 0.67	M = 3.53 ± 0.73
Professionals	M = 3.94 ± 1.02	M = 3.68 ± 0.82

Main effects of training status (first-year students vs. advanced students vs. professionals) on EBs regarding knowledge in physiotherapy and knowledge in medicine.

### Therapeutic health concepts

Hypothesis 3 assumed a main effect of therapeutic health concept regarding a difference between *bps* and *bm*. This assumption was supported by the data. Physiotherapists had higher values in the *bps* (M = 5.56 ± 0.43) than in the *bm* concept (M = 4.11 ± 0.80; t(167) = 21.13, P < 0.001, d_z_ = 1.66).

Hypothesis 4a, which assumed a main effect of training status on the *bps* concept, was predominantly supported by the data (see Table 4). We found significant differences between students and professionals regarding the *bps* concept (F(2, 164) = 5.39, P = 0.005, η^2^_p_ = 0.06). The contrast analysis revealed that the comparison of the first year students with professionals was significant (P = 0.002). Compared to students in the first year, professionals had a more pronounced *bps* concept. Furthermore, the comparison of the first-year students with advanced students was also significant (P = 0.004). Advanced students had a more pronounced *bps* concept than first-year students. However, the comparison of the advanced students with professionals was not significant (P = 0.933).

**Table 4:** **Therapeutic health concepts**

Health concepts	Biopsychosocial concept	Biomedical concept
Training status		
First-year students	M = 5.33 ± 0.47	M = 3.71 ± 0.68
Advanced students	M = 5.61 ± 0.38	M = 3.93 ± 0.64
Professionals	M = 5.61 ± 0.42	M = 4.38 ± 0.85

Main effects of training status (first-year students vs. advanced students vs. professionals) on the biopsychosocial and the biomedical therapeutic health concept.

Hypothesis 4b, which assumed a main effect of training status on the *bm* concept, was not supported by the data, even though training status was related to the *bm* concept (F(2, 164) = 10.99, P < 0.001, η^2^_p_ = 0.12). In fact, we found quite the opposite of what we had expected (see Table 4). The result of the contrast analysis showed that professional physiotherapists had a significantly more pronounced *bm* concept than first-year students (P < 0.001) and advanced students (P = 0.001). The comparison of the first-year students with advanced students was not significant (P = 0.158).

## Discussion

The aim of this paper was to contribute to a better understanding of the health-related EBs and therapeutic health concepts of health care students and professionals. Our findings demonstrate that physiotherapists’ health-related EBs were more sophisticated in relation to knowledge in their own discipline than to knowledge in medicine. At the same time, significant correlations between the domains occurred. So participants with more sophisticated EBs regarding knowledge in physiotherapy had also more sophisticated EBs regarding knowledge in medicine. This dualistic pattern of results may be interpreted as evidence for the co-occurrence of domain-specific and domain-general EBs [6]. In other words, while participants were able to differentiate among different health-related domains regarding their evaluation of knowledge in these domains, they also tended to apply a similar standard to the assessment of knowledge in physiotherapy and in medicine. This suggests that knowledge in physiotherapy and knowledge in medicine have some features in common. It would be interesting to investigate whether similar result patterns can also be found in other health-related domains.

Our results also demonstrate that EBs regarding both physiotherapy and medicine differed between students and professionals. Compared to students, professionals had more sophisticated EBs. This suggests that people’s beliefs about health-related knowledge might have become more sophisticated throughout the course of their professional activities, and that this process is not fully completed after their vocational training. In addition, these findings show that the process of sophistication in developing EBs was not restricted to a person’s own discipline; it appears that this sophistication applied to adjacent disciplines as well. Since their vocational training provides physiotherapy students in particular with physiotherapy-specific knowledge, we may interpret the current empirical result of more sophisticated EBs regarding knowledge in medicine as a transfer effect.

Concerning therapeutic health concepts, we found that physiotherapists were more *bps*-orientated than *bm*-orientated. It would be an interesting challenge for future research to examine the therapeutic health concepts of other health care professionals. It appears plausible, for example, that physicians and medical students may be more *bm*- than *bps*-orientated. There might also be differences in therapeutic health concepts among medical fields, such as higher *bm* and lower *bps* values for cardiologists or radiologists as compared to psychiatrists or general practitioners.

We did not find any higher values in the *bps* concept for professionals compared to advanced students, but did find higher values for advanced students compared to first-year students: It appears that the development of the *bps* concept had already taken place in an early phase of people’s vocational training and remained stable afterward. Advanced physiotherapy students had achieved very high scores in the *bps* concept, which apparently did not allow for further development of this concept throughout their subsequent professional life. So maybe the *bps* concept was already fully developed in advanced students, that is, this concept had already achieved its maximum value during the students’ vocational training. When we look for an explanation for the absent difference between advanced students and professionals, it makes sense to look at what advanced students and professionals have in common and in which aspects they differ from first-year students. A distinguishing characteristic of the school where we recruited our participants is that the students start working with patients (under supervision) in the second half of the first year of training. So in our sample, since all students were surveyed during the first half of the school year, the first-year students would not yet have worked with patients, whereas the advanced students would have. Therefore, exposure to patients could be a relevant trigger for the development of the *bps* concept of physiotherapists. The main goal of health care professionals is taking care of patients, and it appears that contact with patients might be a factor that contributes to the professionalization process.

Professional physiotherapists had a more pronounced *bm* concept than physiotherapy students. It seems that professionalization played a more important role than vocational training in the development of the *bm* concept. A post-hoc explanation of the more pronounced *bm* concept could be that physiotherapists (like most health care professionals) have to deal with diverse professional medical groups (e.g., physicians) in their daily routine. To achieve a common goal (i.e., to cure a patient), mutual understanding and the ability to take other perspectives is necessary [37]. Compared to professionals, physiotherapy students are faced less often with interdisciplinary situations in their vocational training, which may explain why they possess a less developed *bm* concept.

A limitation of the study is that the variability sub-scale of the EBs measure had to be excluded due to psychometric flaws. All findings related solely to the texture sub-scale of the CAEB. Future research should try to include other features of EBs as well. In addition, the generalizability of the results might be reduced by the fact that all students were recruited from only one school of physiotherapy. Further studies in the field might also consider recruiting a broader sample of participants. Finally, the comparison between students and professionals was based on cross-sectional data. This limitation should be addressed in future research by following up on the development of EBs and health concepts in longitudinal studies, or by setting up experimental designs.

Regarding the domain-specificity of EBs that occurred in this study, we also have to keep in mind that different disciplines can be described by different characteristics [38], so it cannot be ruled out that the differences between the physiotherapy and medicine domains can also be explained by idiosyncratic characteristics of those disciplines. These considerations should be addressed in future research where investigators capture the EBs about diverse health-related domains.

## Conclusions

Professionalism is an essential issue in medical education. Different teaching methods (e.g., problem-based learning) have been designed to support students in acquiring professional attitudes [39]. But personal EBs and therapeutic health concepts have been insufficiently considered in these efforts. Accordingly, the current study has tried to shed light on how the occurrence of these concepts compares between students and professionals.

To our knowledge, previous research has not yet explicitly considered differences in personal EBs and therapeutic health concepts between students and professionals. What has been examined, however, is the ‘epistemology’ of students of psychology and medicine [40]. In this research, opposition between a dualistic epistemology (i.e., when people perceive knowledge as an accumulation of unconnected facts) and a relativist epistemology (i.e., when people understand knowledge in relation to the context in which facts are interpreted) was discovered. It was found that 56% of novice medical students were relativists, while 76% of the advanced students were relativists. Psychology students, in contrast, were almost all relativists right from the start (82% of the novice and 93% of the advanced psychology students). This finding is in line with our interpretation that the domain as well as a person’s training status has to be considered in the context of personal EBs [41]. In the same fashion, it was found that most novice medical students started their academic studies with quite simple beliefs, but with more experience modified their understanding of medical knowledge and began to consider it to be less certain and more open to change [3].

Our findings suggest implications for medical education. Learning settings which contain real challenges of professional life may be useful in developing sophisticated handling of domain-specific knowledge. Thus, medical education should try to include challenges of professional life in health care training at an early stage. For example, an interdisciplinary learning context might support the development of health-related EBs in general, and of an integrated therapeutic health concept where people adopt both *bm* and *bps* concepts. We assume that including real challenges of professional life in health care training would enable students to apply various perspectives to health problems and could also facilitate inter-professional work. It seems expedient that interdisciplinary training settings and other real features of professional life should be increasingly assimilated into medical education for health care students. Of course, interdisciplinary training settings are not a panacea: Previous research has found, for example, that the development of EBs requires deeper knowledge in a particular domain [2]. So it cannot be ruled out that interdisciplinary educational situations (in particular when applied in an inappropriate time frame) may overstrain students and result in superficial knowledge about various domains before they have acquired deep knowledge in any particular domain. This, in turn, may interfere with the development of sophisticated EBs. But nevertheless we argue that the benefits of interdisciplinary training would probably outweigh the potential pitfalls. At least in well supervised interdisciplinary settings, reflection about the learning setting can be prompted [42] which in turn may support a growing awareness and the sophistication of EBs as a relevant meta-belief [43] for health care professionals. In addition, students can be supported in understanding the relationships among different disciplines and recognize the pertinent strengths and limitations of the knowledge in the particular disciplines.
